# Efficient TALEN Construction for *Bombyx mori* Gene Targeting

**DOI:** 10.1371/journal.pone.0073458

**Published:** 2013-09-18

**Authors:** Yoko Takasu, Suresh Sajwan, Takaaki Daimon, Mizuko Osanai-Futahashi, Keiro Uchino, Hideki Sezutsu, Toshiki Tamura, Michal Zurovec

**Affiliations:** 1 National Institute of Agrobiological Sciences, Tsukuba, Japan; 2 Biology Centre, Academy of Sciences, and the Faculty of Natural Sciences, University of South Bohemia, Ceske Budejovice, Czech Republic; Technical University of Denmark, Denmark

## Abstract

Engineered nucleases are artificial enzymes able to introduce double stranded breaks at desired genomic locations. The double stranded breaks start the error-prone repair process of non-homologous end-joining (NHEJ), which eventually leads to the induction of mutations at target sites. We showed earlier that ZFNs and TALENs are able to induce NHEJ mutations in the *B. mori* genome. In order to optimize our mutagenesis protocol, we modified one of the reported truncated TALEN scaffolds and optimized it for use in the *B. mori* embryo. We also established a novel *B. mori* somatic cell assay suitable for the preselection of highly efficient TALENs directly in the *B. mori* model system. We compared the efficiency of several TALEN pairs based on three different frameworks using the *BmBLOS2* gene. The new active TALENs show one order of magnitude higher efficiency than those we used previously. We confirmed the utility of our improved protocol by mutagenesis of the autosomal gene, *red egg* (*Bm-re*) and showed that it allows obtaining homozygous mutants in G_1_. Our procedure minimizes the chance of failure in *B. mori* gene targeting experiments.

## Introduction

Engineered nucleases are artificial enzymes able to introduce double stranded breaks (DSBs) at desired genomic locations (for a review see [1]). The occurrence of DSBs eventually leads to mutations at target sites due to errors made in the repair process. Recently, engineered nucleases have been used to induce targeted mutations in a number of species previously inaccessible to reverse genetic methods. This technology opened the way for functional studies as well as for potential commercial applications in a wide range of non-model organisms.

Since the first use of Zinc Finger Nucleases (ZFNs) in 1996 [2] progress has continued to increase dramatically in the development of these engineered enzymes. They were first used in *D. melanogaster* carrying two transgenic constructs expressing ZFN monomers. This protocol involved elaborate steps of making transgenic flies and combining the two transgenic constructs in the same individuals [3]. Subsequent introduction of direct injection of mRNAs encoding chimeric nucleases brought a key simplification and opened up the possibility of using genome targeting for a wide spectrum of species [4]. The introduction of Transcription Activator-like nucleases (TALENs) has recently attracted the attention of a wide scientific community. TALENs seem to be even better than ZFNs because of their simple DNA binding code allowing a high success rate, targeting of a wide range of candidate cleavage sites, and easy modular design of their DNA-binding domains [5].

TALENs are chimeric proteins consisting of an engineered DNA binding domain and a nonspecific nuclease domain. The DNA binding domain is derived from the Transcription Activator-like (TAL) family of bacterial transcription factors of the genus *Xanthomonas*, and the nuclease domain is the Fok I restriction nuclease catalytic domain from *Flavobacterium okeanokoites* [6]. TALENs function in pairs due to the dimerization of the Fok I nuclease domain and they recognize two half sites in the DNA target separated by a 14–30 nucleotide long spacer. The DNA binding domain is made up of a varying number (usually 13–28) of modular repeated units, usually 34 amino acid residues long, each containing two residues crucial for the recognition of a single DNA base [7], [8]. The efficiency of TALENs was reported to be strongly dependent on the optimal truncation of the TAL-effector part of the molecule [9], [10], [11]. The N-terminal TALEN truncations seemed to be limited to 147 N-terminal amino acid residues (NΔ142) preceding the TAL-effector repeats, which were shown to be essential for full DNA binding activity [12], however, Miller et al. [10] reported successful TALENs with 136 N-terminal amino acid residues (NΔ152). The TALENs were further shown to possess high activity for C-terminal truncations of up to 18 amino acid residues (C+18) following the TAL-effector repeats [10], [13]. The C-terminal residues form a linker between the TAL-effector DNA binding domain and the Fok I nuclease domain. TALENs with shorter linkers were shown to have optimal efficiency for DNA targets with half-sites separated by shorter spacers [8].

Several reports have appeared recently describing the use of ZFNs and TALENs in insects, including *D. melanogaster*, *B. mori, Gryllus bimaculatus*, and *Danaus plexipus* [4], [14], [15], [16], [17], [18]. Most of these studies optimized the mutagenesis protocol using a marker gene and assayed the mutants by phenotypic screening. Two studies, however, reported the use of highly efficient ZFNs and mutagenized non-marker genes, including *D. melanogaster* genes *coilin* (*coil, CG8710*) and PAS kinase (*pask, CG3105*) as well as the *D. plexipus* gene *CRY2* [4], [16]. Mutant individuals in these reports were detected solely by molecular methods and were used for further functional analysis. Other reported experiments with ZFNs in *D. melanogaster* also suggest that the coinjection of ZFN mRNAs with a donor plasmid carrying an altered target sequence will allow homologous recombination [4].

*BmBLOS2*, a single gene responsible for epidermal color, was successfully used for the examination of ZFN and TALEN mutagenesis in *B. mori* [15], [19]. The gene is located on the Z chromosome and allows easy detection of G_1_ hemizygous female mutants based on their phenotype. The percentage of yielding founder G_0_ animals (yielders) that transmitted disrupted gene alleles was within the range of 5–30%, the total frequency of mutants was below 5% and varied for different targets [15], [19]. These experiments showed that both ZFNs and TALENs can be used for gene targeting in the silkworm.

As the next step towards the use of engineered nucleases for functional gene analysis it is important to reduce the amount of labor associated with DNA-based screening, which is needed for the mutagenesis of genes with unknown phenotypes. Here we show a further optimization of the *B. mori* NHEJ mutagenesis protocol. We developed an easy somatic cell assay based on embryo microinjection to evaluate the newly constructed TALENs and substantially increased the efficiency of mutagenesis by optimizing the truncated TALEN framework. We confirmed the utility of our improved protocol by disrupting an autosomal gene called *red egg* (*Bm-re*) encoding a transporter involved in the ommochrome biosynthesis pathway [20]. The optimizations presented in this report significantly decrease the chances of failure in *B. mori* targeted mutagenesis.

## Materials and Methods

### Silkworm strains

Two nondiapausing *B. mori* strains were used for targeting, *w1*-*pnd* (*white egg 1*) for *BmBLOS2* (*distinct oily*; Z-49.6), and *pnd* for *Bm-re* (*red egg*; 5–31.7). The strains were maintained at the Transgenic Silkworm Research Unit (National Institute of Agrobiological Sciences, Tsukuba, Japan). The larvae were kept on an artificial diet (Nihon Nosanko, Yokohama, Japan) at 25°C.

### Framework plasmid construction

**Figure 1:**
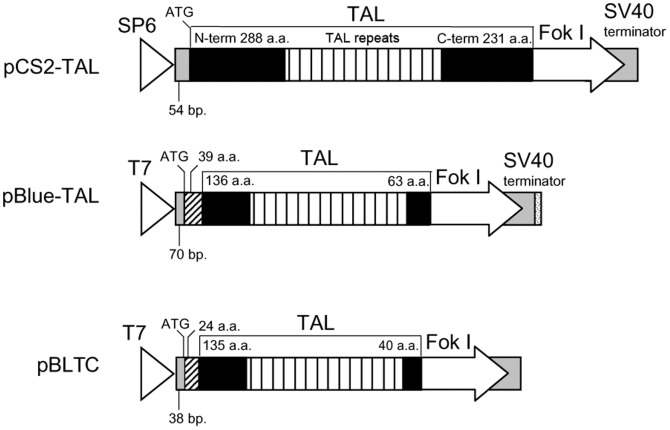
Schematic representation of plasmid constructs. Plasmid pCS2-TAL, pBlue-TAL and pBLTC contain a central region of 34 a.a. repeats (vertical hatch) flanked by N- terminal and C-terminal TAL sequences of different lengths (solid rectangles). SP6 and T7 indicate the SP6 and T7 promoters (open arrowheads), respectively. Arrows represent the *Fok* I nuclease domain, gray rectangles the 3′ and 5′ UTRs and rectangles with angled hatch the sequences encoding nuclear localization signals.

Construction of a pCS2-TAL (see Fig. 1) used for the expression of BLT TALENs was described earlier [19]. TALEN constructs for the production of pBLTC TALEN was obtained from Cellectis Bioresearch (Paris, France).

Our framework plasmid used for the expression of BLTS TALENs was based on the scaffold (NΔ152/C+63), described by Miller et al. (2011). It lacked 152 N-terminal amino acids and we added 39 amino acids (originally from pVAX-based vector) required for nuclear localization to remaining 136 amino acids of the TAL N-terminus (Fig. S1A and B). To improve the compatibility of the synthesized RNA with *B. mori* translation machinery, we optimized the codon usage (silkworm genome project), added a Kozak sequence and a short 60 bp 5′ UTR track from the *B. mori* actin gene, as well as an SV40 3′ UTR and a short poly(A) stretch on the 3′ end of the transcribed part of the chimeric construct. (Fig. 1). We also added T7 promoter and made the scaffold compatible with the Addgene kit (Addgene, Cambridge, MA, USA) for Golden Gate assembly [5] by including *Esp* 3I restriction sites (Fig. S1). The construct was synthesized by GenScript (Piscataway, NJ, USA). The DNA fragment was obtained in pUC57 and digested with *Eco*R I and *Stu* I, treated with Mung bean nuclease (Takara-bio, Kyoto, Japan) and subcloned into a pBluescript KS+ plasmid digested with *Pvu* II. The resulting plasmid was denoted as pBlue-TAL (Fig. 1).

### DNA target site selection and Golden Gate TALEN assembly

**Figure 2:**
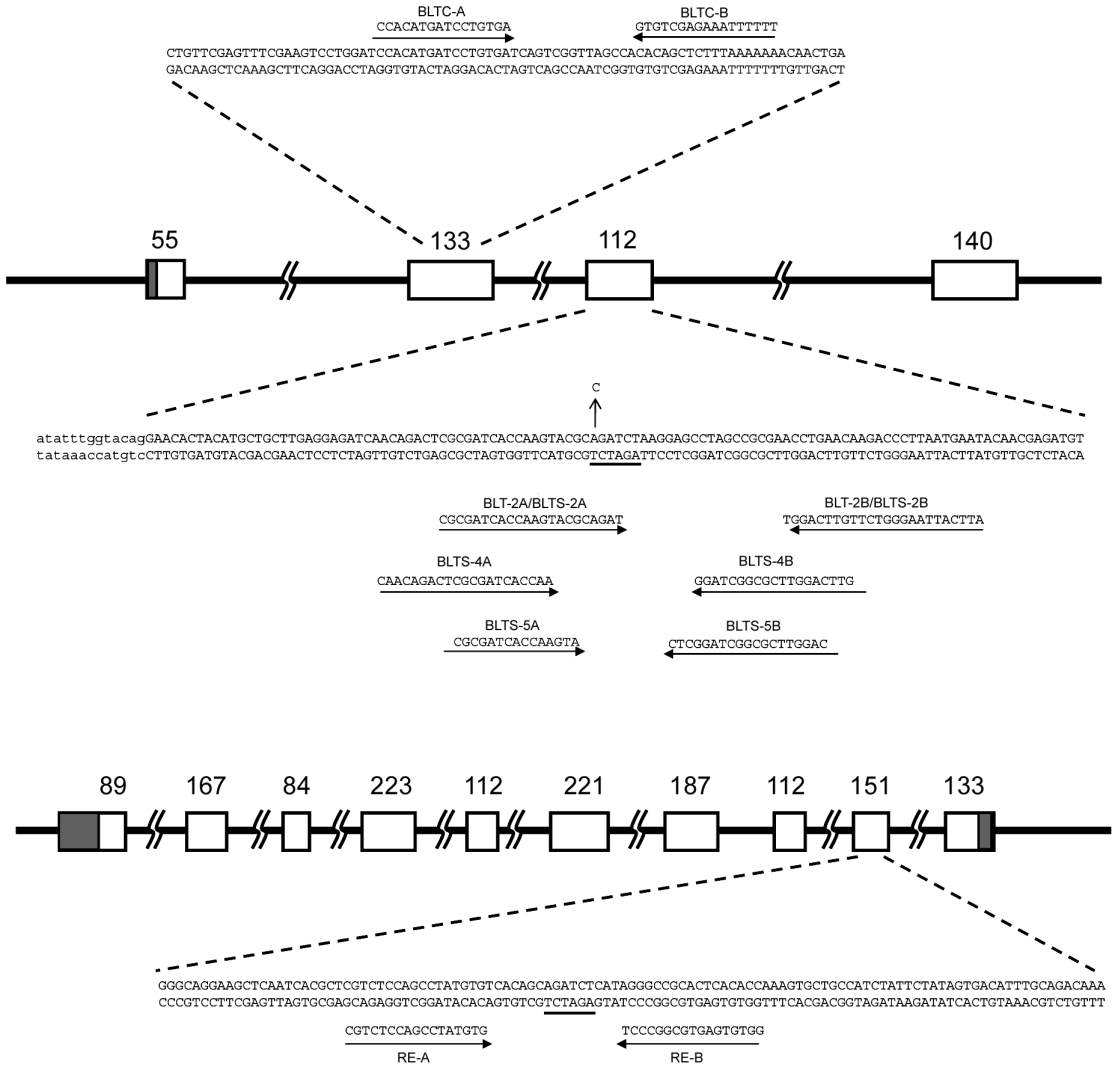
TALEN targets. (A) Positions of TALEN targets in the *BmBLOS2* gene. Open rectangles and numbers above represent exons and their length in bp. The filled box is the 5′- UTR. The sequence above the line indicates part of exon 2 with BLTC target sites and their names. The sequence below the line indicates the third exon with BLT-2 and BLTS targets. *Bgl* II restriction site is underlined. The c above the Bgl II site indicates polymorphism in *w1-pnd* strain. (B) Positions of TALEN targets in the *Bm-re* gene. Open rectangles and numbers above represent exons and their lengths in bp. Filled boxes are 5′-and 3′ UTRs. The sequence below the line indicates the ninth exon (capital letters). *Bgl* II restriction site is underlined.

The DNA targets for TALENs were searched for using the web program TALEN targeter [21] (https://boglab.plp.iastate.edu/). Four TALEN half sites were selected in the third exon of the *BmBLOS2* gene; two half sites were located on each side of the internal *Bgl* II site for easy evaluation of TALEN efficiency (Fig. 2A). A partial overlap of both targets allowed combinations of individual TALEN monomers into 4 different TALEN pairs named BLTS-4-7 (Table 1) which had spacer lengths of 18, 15, 15 and 12 nt, respectively. A comparison of the efficiency of BLTS-4-7 was used to determine the optimal spacer length. The selection of BLT-2 (BLTS-2) target site was described earlier [19]. The target site for the *Bm-re* gene was selected in the ninth exon (Fig. 2B), since the known viable mutation in this gene was localized to the same exon [20]. The target for the TALEN BLTC was in the second exon of *BmBLOS2* (Fig. 2A). The design, synthesis and validation of BLTC TALEN pair were performed by Cellectis Bioresearch (Paris, France).

**Table 1:** Summary of TALENs used in this study.

Target	TALENs	N-terminus (a.a.)	C-terminus (a.a.)	Recognition site (bp)		Spacer (bp)
				Left	Right	
BLT-2	BLT-2A, BLT-2B	288	231	22	24	19
BLTS-2	BLTS-2A, BLTS-2B	136	63	22	24	19
BLTS-4	BLTS-4A, BLTS-4B	136	63	21	19	18
BLTS-5	BLTS-4A, BLTS-5B	136	63	21	19	15
BLTS-6	BLTS-5A, BLTS-4B	136	63	15	19	15
BLTS-7	BLTS-5A, BLTS-5B	136	63	15	19	12
BLTC	BLTC-A, BLTC-B	135	40	16	16	15
RE*	RE-A, RE-B	136	63	17	17	16

* Target for this TALEN pair is in the *Bm-re* gene, all other TALENs target the *BmBLOS2* gene.

New BLTS TALEN sequences were first designed *in silico* by combining the appropriate repeat segments specific to the DNA targets (selected by TALEN targeter as described above). DNA constructs containing the segment arrays were then prepared by Golden Gate assembly [22] using the Golden Gate TALEN Kit from Addgene and inserted into the scaffold plasmid pBlue-TAL described above. The constructs contained the repeat modules with four different RVDs (NI, HD, NN, NG). The details of new TALEN pairs are shown in Table 1.

### *in vitro* mRNA synthesis

The *pBlue-TAL and BLTC e*xpression plasmids with different TALEN constructs were purified with a HiSpeed plasmid midi kit (Qiagene, Dusseldorf, Germany), linearized by digestion with *Xba* I or *Hind* III, and treated with proteinase K (Nakarai, Japan). Capped and polyadenylated RNAs were then synthesized using an mMESSAGE mMACHINE T7 Ultra kit (Ambion, Carlsbad, CA, USA) as described previously [17]. The TALEN mRNAs were precipitated with LiCl, and washed three times with 70% ethanol.

### Silkworm embryo microinjection

The capped and poly(A)-tailed mRNAs encoding TALEN pairs were dissolved in 0.5 mM phosphate buffer (pH 7.0) to give a final concentration of 0.5 µg/µl for each mRNA except for the BLTC TALEN mRNA, which was used for germline targeting at a concentration of 0.2 µg/µl. A total of 3–5 nl of RNA solution was injected through the chorion into silkworm embryos at the syncytial preblastoderm stage (4–8 hrs after oviposition) as described previously [17]. The small hole in the chorion caused by microinjection was sealed with cyanoacrylic glue (Aron Alpha, Konishi Co, Osaka) and the embryos were incubated at 25°C in a humidified atmosphere.

### Somatic cell assay based on *B. mori* embryo microinjection

**Figure 3:**
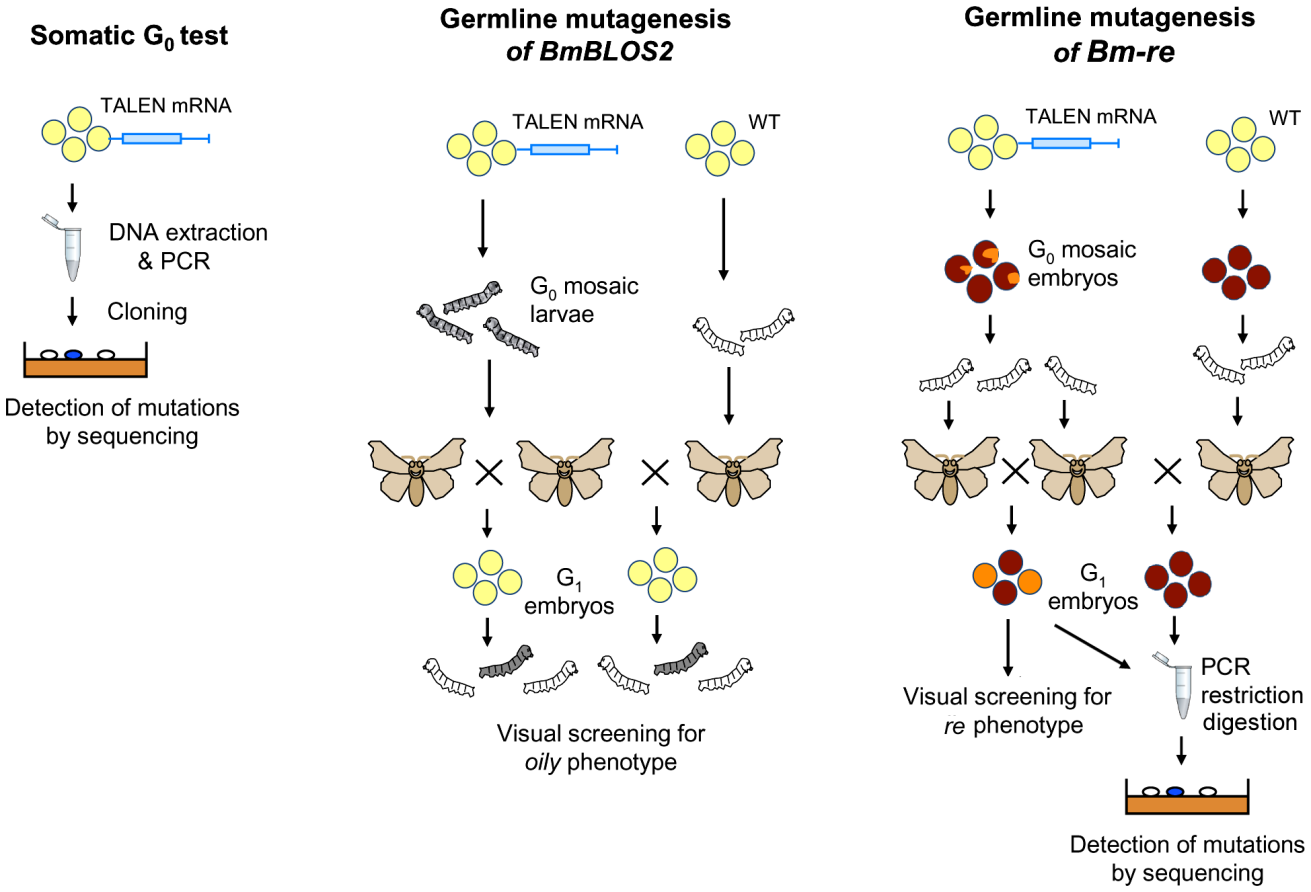
Comparison of somatic cell test and germline targeting experiments in silkworms. (A) In the somatic cell test the mutagenized G_0_ embryos were used for determination of frequency of mutated alleles by DNA analysis. (B) In the *BmBLOS2* experiment (using *w1-pnd Bombyx* strain) the G_1_ individuals were screened for an *oily* phenotype. In *Bm-re* (*RE*) mutagenesis (using *pnd* strain) the frequency of mutant alleles was tested in G_1_ individuals by DNA analysis.

To test the ability of novel TALEN pairs to induce the NHEJ mutations at target sites, we injected RNAs (0.5 µg/µl) for individual TALEN pairs into the early embryos, incubated them for three days in a humidified atmosphere, isolated genomic DNA from pooled samples and determined the frequency of mutant alleles (Fig. 3A). To lower the bias from individual microinjections we microinjected 90–100 embryos for each of the TALEN pairs. The embryos were scored for viability as indicated by green autofluorescence under UV excitation. Genomic DNA was isolated from a pooled sample of 30–45 living embryos using the Tissue Protocol, Blood and Tissue Genomic DNA extraction miniprep system (Viogene). The targeted DNA region was amplified by PCR and the PCR fragments were subcloned into bacteria and sequenced. These experiments showed that for the novel, highly efficient TALENs it was sufficient to analyze 45–50 randomly picked bacterial colonies to obtain several mutant clones.

### Crossing scheme and screening strategy

Mutagenized G_0_ individuals were screened for mosaicism (patches of *oily* cuticule on the WT background for *BmBLOS2* or yellow spots on the embryos for *Bm-re*). Since the *BmBLOS2* gene is located on the *Z* chromosome, we crossed the mutagenized G_0_ male moths to mutagenized or WT females (Fig. 3B) as described earlier [19]. The G_1_ offsprings (usually hemizygous females) were screened for the *oily* phenotype. Mutations were confirmed by PCR amplification and sequencing of the targeted product.

The crossing scheme for mutagenesis of the autosomal *Bm-re* gene was the same as for the sex-linked gene *BmBLOS2*, except that screening for phenotype was limited because we could not take advantage of female hemizygosity (Fig. 3C). Consequently, PCR screening had to be used as the principal method to detect mutations in the G_1_ heterozygotes as phenotypic screening could only detect the rare mutant homozygotes obtained from a sibling G_0_ cross.

### G_1_ brood sampling

To estimate the mutant allele frequency in G_1_ “G_1_ brood sampling test” was performed. About 25% of the eggs from each brood were pooled for genomic DNA extraction, using a Blood and Tissue Genomic DNA extraction miniprep system (Viogene, Sijhih, Taiwan). the targeted region was amplified by PCR using KOD FX Neo Polymerase (Toyobo, Osaka, Japan). The PCR products were subcloned into the pTA2 vector using a Target Clone-Plus kit (Toyobo). The ligation mix was used for the transformation of *Escherichia coli DH5α* and plated on ampicillin dishes containing X-gal. Individual inserts from 44 white colonies were sequenced to check for modification of the target sequence.

To detect mutant-yielding broods, another variant of G_1_ brood sampling was performed. Two batches of 25 eggs were collected from each brood and genomic DNA was extracted. The target region was amplified with PCR and the products were treated with a restriction enzyme *Bgl* II.

### Sequence analysis

Genomic DNA was isolated using the Blood & Tissue Genomic DNA Extraction Miniprep System (Viogene) as described above. Up to 30 mg of tissue was used for each DNA sample. DNA fragments containing the targeted sites of interest were amplified by PCR using specific primers designed from flanking regions. The *BmBLOS2*-specific primers F1 (5′-CTTCCAATTTGAGGGCAATG-3′) and R1 (5′-AATTTCACCACCTCATTCAACT-3′) were used previously with BL-1 ZFNs [17]. The primers for BLTC screening were as follows: F1: TGGTCCAGTAGGTTTGAAGTA R1: GACAATGAAGCTTCTCAGATGA. The primers for *Bm-re* screening were: reF1: GTCATCTGGTATGGTGGTCAG and reR1: ATAAATGACGGTAGCTACTGTC. The PCR products were gel purified and sequenced using the same primers.

### Detection of mutants by multiplex colony PCR

The DNA fragments containing the targeted sites of interest from the previous paragraph were either sequenced as described above or analyzed by multiplex PCR. Aliquots of white bacterial colonies from X-gal ampicillin LB plates containing pTA2 clones were directly resuspended in PCR buffer, mixed with a series of 4 primers and used for amplification. We expected 3 PCR products for the wild-type sequence: 346 bp, 216 bp and 172 bp. The absence of one or both shorter PCR bands in the electrophoretogram indicated a mutation. The primers for the multiplex PCR screening of the BLTS-4-7 mutants were: pAT2.F GTAATACGACTCACTATAGGGC, pAT2.R – ACAGGAAACAGCTATGACCATG; Ex3Bgl.De - CGATCACCAAGTACGCMGATCT, Ex3Bgl.R GGTTCGCGGCTAGGCTCCTT). The BLTC primers were: pAT2.F - GTAATACGACTCACTATAGGGC, pAT2.R - ACAGGAAACAGCTATGACCATG, Dm.multi.F - CATGATCCTGTGATCAGTCGGTT, Dm.multi.R – GAGCTGTGTGGCTAACCGAC.

## Results

### Construction of new TALENs

The TALENs used in earlier experiments targeted the third exon of *BmBLOS2* gene located on the Z chromosome [15], [19]. The TALENs showed a high success rate since all of them produced mutations, but the efficiency of NHEJ mutagenesis was still relatively low – below 5%. Since the truncation of the TAL effector was reported to increase the TALENs' activity, we modified the framework described by Miller et al. [10], which contained a truncation of the TAL DNA binding domain to 136 amino acids at the N-terminus and 63 amino acids at the C-terminus. We also added 39 amino acid long N-terminal sequence containing nuclear localization signal (Fig. 1; we refer to it as an NΔ152/C+63 scaffold in further text). The appropriate constructs encoding individual TALENs were prepared by Golden Gate assembly.

### Somatic cell/embryo injection assays of TALEN activity

Since there are large differences in efficiency among the newly designed TALENs we developed a simple assay based on microinjection of early *B. mori* embryos with TALEN RNAs and examining the frequency of mutant alleles from pooled DNA samples (see Material and methods). The assay is based on *B. mori* genomic DNA analysis and therefore independent of prior knowledge of phenotypes of relevant genes. The details are shown in Fig. 3A.

To examine the utility of this novel test we assayed the efficiency of the BLTS-4-7 TALENs. We microinjected the mRNAs for individual TALEN pairs into 90–100 *B. mori* embryos, and after 3 days only living embryos assayed by UV autofluorescence were used for genomic DNA isolation. The targeted region was amplified by PCR, subcloned into a bacterial plasmid, and checked for modification of the target sequence. The results of the mutagenesis are summarized in Table 2. Among 44–48 clones examined for each TALEN the frequency of detected mutant clones ranged from 0 to 47.9%. No mutations were detected among the 44 BLTS-7 clones examined, suggesting that this TALEN was not functional. BLTS-6 displayed the highest activity with 23 mutant clones out of 48 clones examined (47.9%), whereas BLTS-4 and BLTS-5 TALENs produced 3 and 6 mutant clones out of 46 (6.5 and 13.0%), respectively. The individual sequences of target areas are shown in Fig. 2.

**Table 2:** Efficiency of TALENs in *BmBLOS2* mutagenesis estimated by a *B. mori* somatic cell assay.

Target	Sequenced clones	Mutant clones	Efficiency (%)
BLT-2	40	1	2.5
BLTS-2	45	8	17.8
BLTS-4	46	3	6.5
BLTS-5	46	6	13.0
BLTS-6	48	23	47.9
BLTS-7	44	0	0.0
BLTC	45	5	11.1

We also compared BLT-2 and BLTS-2 TALENs. The BLT-2 TALEN pair, which was used previously (15), contained a full length framework (N288/C+231). The TAL repeated units from the full length framework were recloned into our new truncated NΔ152/C+63 framework and designated as BLTS-2. The use of both TALEN pairs side by side in the same experiment allowed direct comparison of both TALEN frameworks. As shown in Table 2, among the 40 clones examined from the BLT-2 somatic cell assay, only a single (2.5%) mutant clone was detected compared to 8 (17.8%) mutants among the 45 clones examined utilizing the same assay with BLTS-2.

The detection of mutations by sequence analysis of PCR fragments amplified from bacterial clones worked well, but our alternative method based on restriction digestion of PCR product failed in this particular batch of *B. mori* embryos, because of high polymorphism in the first 5′ adenine position of the *Bgl* II site. Therefore, we also tested the utility of multiplex PCR for mutant detection. The results are shown in Fig. S2. As is evident, we were able to detect at least 81% of the mutants by multiplex PCR, thus confirming that this method may be used as an alternative approach for mutant detection.

### High-efficiency germline targeting of the *BmBLOS2* gene using TALENs based on the NΔ152/C+63 framework

To test the function of novel TALEN pairs in germline mutagenesis we microinjected 191–192 embryos with mRNAs encoding the BLTS-4-7 and BLTS-2 TALEN pairs described above and compared the results with previous reports.

**Figure 4:**
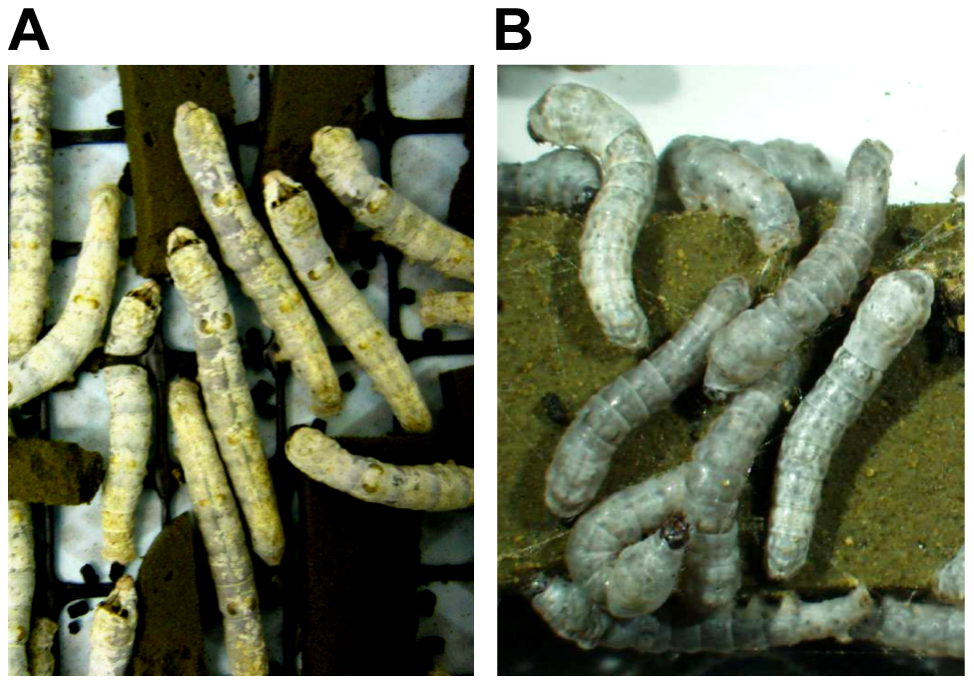
Somatic and germline mutations of *BmBLOS2* in silkworm larvae. (A) Fifth instar G_0_ larvae containing mosaic areas of *BmBLOS2* mutant tissue (B). Third instar G_1_ larvae with *oily* mutant phenotype (marked by arrowheads).

As shown in Table 3, the hatchability of embryos was 31.89–52.8%. The frequency of somatic mosaics for BLTS-4,5 and 6 and BLTS-2(NΔ152/C+63) reached 81.8–100% with the proportion of *oily* epidermis within the mosaic animals being quite high, sometimes covering most of the body (Fig. 4). We did not check the difference between males and females, but at least for the injection with BLTS-6 the efficiency was so high that all males and females displayed *oily* mosaicism. In contrast, the larvae from embryos microinjected with BLTS-7 did not show any mosaicism, thus confirming that this combination of TALENs was inactive.

**Table 3:** Efficiency of TALENs in *BmBLOS2* mutagenesis evaluated by the *oily* phenotype.

Target	Eggs injected	% hatched	% somatic mosaics (G_0_)	% yielders	% germline mutants (G_1_)
BLT-1*	480	36.7	15.3	10.7	0.7
BLT-2*	478	20.9	9.5	6.3	0.5
BLTS-2	192	52.1	84.9	78.6	3.0
BLTS-4	191	31.9	81.8	100.0	10.4
BLTS-5	191	38.2	97.5	100.0	10.7
BLTS-6	191	50.8	100.0	100.0	49.9
BLTS-7	192	42.2	0.0	5.0	0.2
BLTC	432	54.9	87.8	39.0	4.4
B2**	968	23.4	46.0	31.0	Not shown

* Results from Sajwan et al. 2013.

** Results from Ma et al. 2012.

As described in Material and methods and shown in Fig. 3B the G_0_ males that emerged from microinjected embryos were crossed either with the sibling G_0_ or with WT females and the resulting progeny were scored for the *oily* phenotype. The numbers of *oily* mutants are shown in Table 3. As indicated, yielding founder G_0_ animals (yielders) for the G_0_ moths injected with BLTS-4-6 reached 100%.

**Figure 5:**
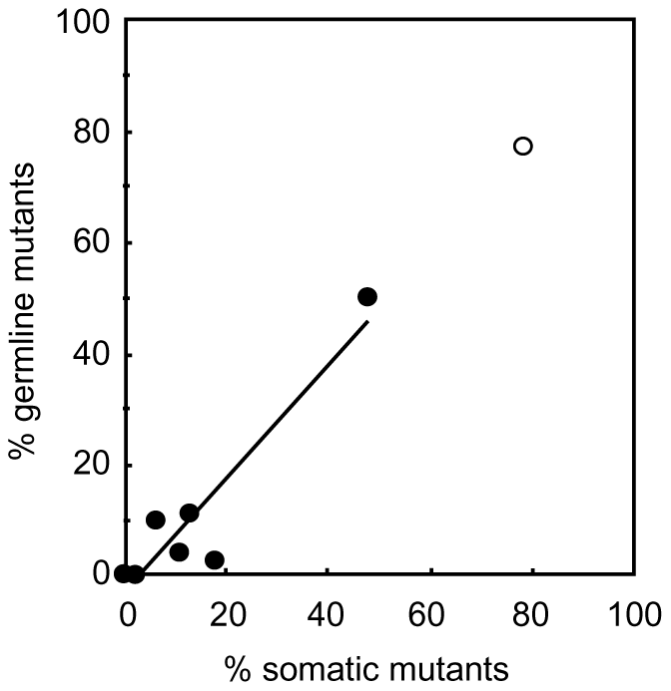
Correlation between the results from a somatic cell test and frequency of G_1_ mutants in *B. mori* based on data from mutagenesis with BLTS and BLTC and *Bm-re* TALENs. The number of BLTS and BLTC mutants is underestimated due to a lower number of males detectable by phenotypic screening. Solid circles represent BLTS, BLT-2 and BLTC, whereas the open circle denotes the *Bm-re* TALEN.

The proportions of G_1_ *oily* mutants induced by BLTS-4-6 and detected in the total progeny was 10.4%, 10.7% and 49.9%, respectively. For comparison, the microinjections with BLTS-2(NΔ152/C+63) and BLTS-7 TALENs resulted in the appearance of 3% and 0.2% *oily* mutants, respectively. The proportions of G_1_ mutants obtained for individual TALEN pairs correlated well with the results from both the somatic cell assay and the occurrence of G_0_ mosaics (Fig. 5). For highly efficient TALEN pairs, the efficiency of G_0_ mosaics reached nearly 100%. Although we could not measure the area of *oily* skin, it also appeared to be roughly proportional to the efficiency of G_1_ mutagenesis and the somatic cell assay. Except for BLTS-7, the frequency of mutants obtained with the new TALENs confirmed a very high efficiency of our novel TALEN scaffold.

### Germline targeting of the *BmBLOS2* gene using a TALEN pair based on the NΔ153/C+40 framework

**Figure 6:**
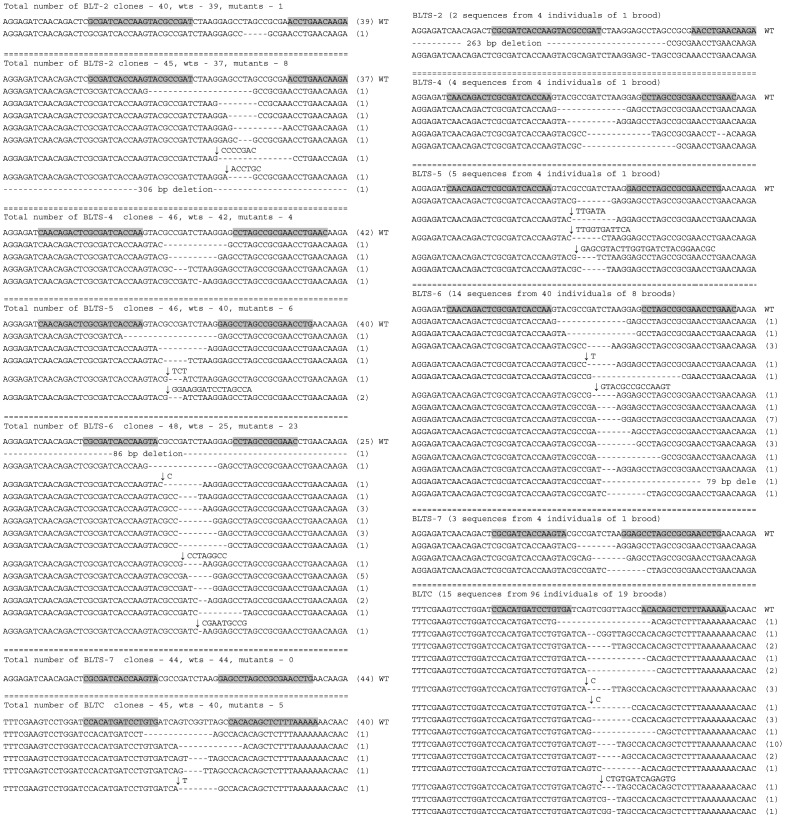
TALEN-induced insertions/deletions in *BmBLOS2*. (A) Mutant alleles obtained from the somatic assay; (B) Mutant alleles obtained from G_1_ individuals. Sequences of mutant alleles are aligned with the respective wild-type sequences. TALEN target sites in the wild-type sequences are highlighted. The number of individuals carrying identical mutation is indicated to the right of each sequence.

In order to compare the efficiency of various TALEN pairs for mutagenesis we also included a TALEN pair containing an N-terminal truncation of 135 amino acid residues preceding the TAL-effector repeats and a C-terminal truncation of 40 residues following the TAL-effector repeats. This TALEN pair was designated as BLTC (NΔ153/C+40). Its target was located in exon 2 of the *BmBLOS2* gene, and contained a 15 nucleotide long spacer (Fig. 2A). We first tested the efficiency of BLTC in our somatic cell assay. The results are shown in Table 2. We found 5 mutated clones among the 45 examined (11.1%). Next, we performed somatic and germline mutagenesis and screened for *oily* phenotype; the results are shown in Table 3 and Fig. 6. The BLTC TALEN produced 87.8% of mosaics in G_0_ and 4.4% *oily* G_1_ mutants. These data show that although the BLTC TALEN had similar efficiency in the somatic cell test and displayed a similar frequency of G_0_ mosaics compared to the BLTS-4-6 TALENs, it induced a 4.4–11.3-fold lower frequency of germline mutations in G_1_.

### Mutagenesis of the autosomal egg color gene *Bm-re*

**Figure 7:**
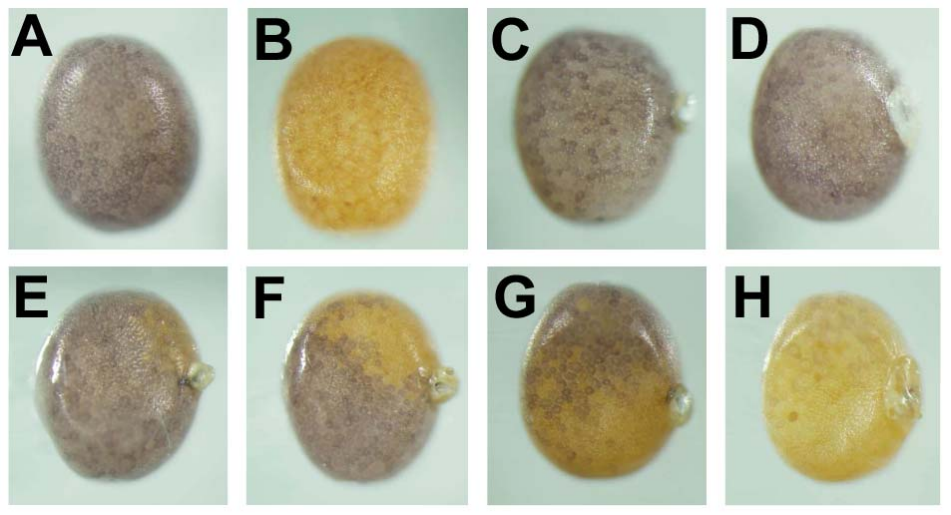
Mutagenesis of the *Bm-re* gene. Embryos were photographed five days after *Bm-re* TALEN microinjection. (A) not injected control, (B) *Bm-re* mutant, (C, D) embryos injected with buffer, (E-H) G_0_ embryos injected with TALEN mRNA.

**Figure 8:**
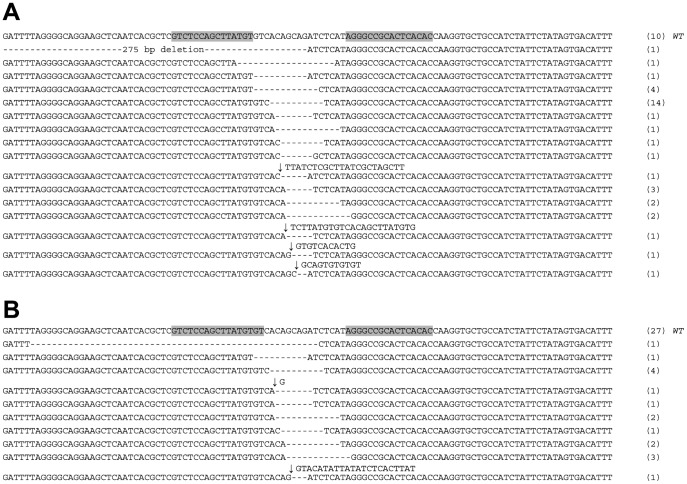
Alignment of mutated *Bm-re* alleles obtained from sequence analysis of G_0_ and G_1_ individuals. The number of individuals carrying identical mutations is indicated to the right of each sequence. TALEN target sites in the wild-type sequences are boxed. (A) Mutant alleles from the somatic assay; (B) G_1_ germline mutations.

So far all of the successful experiments with gene targeting by chimeric nucleases in *B. mori* were performed on a single gene, *BmBLOS2*, which is localized on the Z chromosome. To test the efficiency of our truncated TALEN (NΔ152/C+63) framework on an autosomal gene, we chose the gene *red egg* (*Bm-re*) which is responsible for the color of the egg serosa. Whereas the wild type egg color is dark brown, the known mutant allele of the *Bm-re* gene described earlier [20] is characterized by an orange egg color. The mutant phenotype would, however, be visible only in homozygous *Bm-re*/*Bm-re* individuals (Fig. 7) requiring additional sibling crosses; therefore, we determined to detect mutant alleles by molecular analysis. A single TALEN pair was selected to target the position containing a 16-bp spacer with a *Bgl* II site within the 9th exon of the *Bm-re* gene (Fig. 2B). First we performed the somatic cell assay as described in Material and methods. The results are shown in Table 4 and Fig. S3. We found that 36 out of 46 clones examined (78.3%) carried mutations. The sequences of mutant *Bm-re* clones are shown in Fig. 8A.

**Table 4:** Efficiency of TALENs in *Bm-re* mutagenesis.

Target	% mutants in embryos	% somatic mosaics	% yielders	% germline mutant alleles*
RE	78.3	72.4	100.0	77.3

* Calculated from the PCR analysis results of 8 broods.

The high efficiency of the new “*Bm-re*” TALEN pair was also confirmed by a high frequency of G_0_ mosaics and through G_1_ mutants. We injected 192 embryos and the hatchability was 45.8%. As shown in Table 4, 72.4% of the injected G_0_ embryos displayed dramatic somatic mosaicism. The extent of mosaicism differed among the embryos from relatively small patches in close proximity to the injection point to large areas covering almost the entire embryo (Fig. 7). Since the capacity of rearing was limited, only a part of G_0_ silkworms were reared to evaluate mutagenesis efficiency in G_1_. We obtained one G_1_ brood by sibling mating of mutagenized G_0_ individuals and 8 G_1_ broods by mating of G_0_ males with the WT females. Nine broods of fertile G_1_ eggs were obtained. Only one of the broods was obtained by sibling mating of mutagenized G_0_ individuals; 8 other broods were produced by mating of G_0_ mosaic males with WT females. All of the individuals received from the first (sibling mating) brood showed a *Bm-re* phenotype. To estimate the approximate frequency of mutated alleles in the 9 broods a part of eggs were sampled to detect mutations in the target site. Genomic DNA was extracted from two batches of 25 eggs from each brood, and the target region amplified by PCR was digested with the restriction enzyme *Bgl* II. As expected, the PCR product specific for the single brood obtained by sibling mating contained only the mutated allele, which confirmed the efficiency of mutagenesis of this brood was 100% in this experiment. In the other broods we detected both the wild-type and mutated PCR products (Fig. 8B and S3). This meant that all microinjected G_0_ parents produced a considerable number of mutated germ cells. To estimate the total efficiency of mutagenesis in the remaining 8 broods we determined the proportion of mutant alleles among the cloned PCR products of the amplified target areas. As shown in Table 4, we found 17 mutations among the 44 clones analyzed (38.6%), suggesting that the eight G_0_ males used for crosses with WT females carried 77.3% of gametes with mutated *Bm-re* alleles (the frequency of mutated *Bm-re* alleles in G_0_ male gametes was two times larger than the rate of mutated alleles detected in G_1_ individuals since the WT females did not contribute to this number).

These data showed that the “*Bm-re*” TALEN also had very high mutagenic efficiency, comparable to or perhaps even higher than the BLTS-4-6 TALENs.

## Discussion

To minimize the chances of failure in *B. mori* gene targeting experiments we present here the optimization of the NHEJ mutagenesis protocol. It includes the establishment of a somatic cell assay suitable for the preselection of highly efficient TALENs directly in the *B. mori* model system, the optimization of the TALEN scaffold, and the application of PCR in place of mutant phenotype screening.

Gene targeting experiments have several intrinsic problems which cannot be fully addressed in heterologous systems and are hard to predict; these include off-target effects and toxicity of the engineered nuclease, DNA polymorphism in the targeted area, unfavorable chromatin structure, and uncontrolled DNA modification. In order to preselect the most suitable TALENs for germline targeting experiments, we introduced a simple somatic cell assay based on *B. mori* embryo microinjection. This assay is very close to the final experimental conditions, so that it is sensitive to most of the negative influences mentioned above. In addition, it is based solely on DNA analysis and independent of prior knowledge of the phenotype of the targeted gene. Our results showed a very good correlation between the somatic cell/embryo microinjection test and the efficiency of germline mutagenesis (Fig. 5). An advantage of obtaining direct information about the function of an engineered nuclease from the same model system has been used in mammals in the form of various tissue-culture assays [23], [24]. Embryo-based somatic assays for TALENs were also recently established for zebrafish and cattle [25], [26].

Besides the enzymatic activity of engineered nucleases, other factors may also contribute to the efficiency of mutagenesis. It has been known for many years that, in addition to the 5′ cap and a poly(A) tail, the presence and sequences of 3′ and 5′ UTRs play important roles in the initiation of translation and RNA stability [27]. We propose that the high activity of our new TALEN pairs was reached on account of two key factors in the design of the NΔ152/C+63 scaffold: the truncation of TAL ends and the optimization of the transcribed part of the construct for the *B. mori* translation machinery.

The use of *BmBLOS2* as a model gene in *B. mori* targeting experiments allowed the comparison of different scaffolds and approaches. Although in most of our experiments we did not directly compare the TALEN activity solely on the basis of the framework, the increase of efficiency of the BLTS TALENs compared to nucleases in earlier reports is clear. We also have to keep in mind that the number of G_1_ individuals carrying the *BmBLOS2* mutation is larger than the numbers shown in Table 3 since our screen covered only the individuals with an *oily* phenotype and the heterozygous males were not detected.

The high efficiency of BLTS-4-6 TALENs in our experiments surpassed the activity of all TALENs so far examined in *B. mori*, including the BLTC TALEN pair (carrying the NΔ153/C+40 framework) reported here, the BLT1-3 TALENs used in our earlier study [19] representing a full-length TAL-effector (N288/C+231) or the TALENs based on NΔ48/C+63 framework (containinng 240 N-terminal amino acids from TAL-effector) used in the earlier report of Ma et al. [15]. As shown in Table 3, the frequency of yielders from TALENs carrying the 63 C-terminal amino acid residue framework used in that study [15] reached 11–31%. The use of TALENs made in the (N288/C+231) framework in our previous study [19] produced 6–11% of yielders. In contrast, our BLTS-4-6 and “*Bm-re*” TALENs prepared in the new NΔ152/C+63 truncated framework showed 100% of yielders. For comparison, the most efficient ZFN used in *D. melanogaster*, the *rosy ZFN*, produced 41% of yielders and 8.2% of G_1_ mutants [4].

The parallel mutagenesis experiments with BLTS-2 and BLT-2 allowed a direct comparison of the (N288/C+231) and (NΔ152/C+63) scaffolds, respectively. There was, however, a highly polymorphic DNA binding site for BLT-2/BLTS-2 in the *w1-pnd* embryos used in this study which negatively affected the efficiency of BLTS-2 (NΔ152/C+63) TALENs and probably reduced the observed efficiency of this TALEN in a portion of the *w1-pnd* silkworms used in these experiments. Despite this negative bias, the frequency of BLTS-2 (NΔ152/C+63) mutants was still approximately six times higher than the mutant frequency induced by BLT-2 (N288/C+231).

Our study also uncovered important information regarding the influence of spacer length on TALEN activity. The BLTS-4-7 TALEN pairs were prepared through the combination of 4 TALEN monomers, this yielded pairs containing spacers with lengths from 12 to 18 bp (Table 1). BLTS-7 had the shortest spacer of 12 bp, which was below the recommended limit [8]. Consistently, Christian et al [8] observed that the (NΔ152/C+63) framework TALEN pair with a 12 bp spacer showed less than 25% activity of the optimal control [8]. In our experiments BLTS-7 showed almost no activity despite the fact that both individual TALEN monomers from the BLTS-7 pair displayed high efficiency in the BLTS-5 and -6 TALEN pairs (Table 1 and 3). This confirmed that the 12 nucleotide-long spacer is not sufficient for the activity of a truncated TALEN containing 63 amino acids downstream of the repeat array.

Our experiments suggest that there are large differences in mutant frequencies among different broods. Out of the 30 broods that we received in the experiments with BLTS-6, we observed five G_1_ broods consisting of only mutants and three broods where the *oily* mutants accounted for the majority of the progeny (data not shown). If we are able to detect such broods with a high proportion of mutants by a simple G_1_ sampling assay (as shown for “*Bm-re*” TALEN mutagenesis), it will be possible to use the progeny of such broods for further crossings to obtain homozygous mutants.

The improved TALEN mutagenesis protocol described here is a big step towards the routine use of gene targeting in *B. mori*. The improved TALEN framework and the increased efficiency of mutagenesis greatly reduce the amount of labor needed for the detection of mutations in genes with unknown phenotypes. We have also developed a somatic cell assay for evaluation of newly constructed TALENs showing a strong correlation with the results from germline mutagenesis despite the underestimation of mutant allele numbers due to the inability to detect heterozygous males by phenotypic screening. Similarly, a “G_1_ brood sampling test” based on DNA analysis was introduced in order to estimate the frequency of mutations in G_1_ and to detect broods with a high proportion of mutants. The use of such broods for further experiments will enable easy and fast establishment of homozygous mutant strains. We showed for the first time that the new protocol can be applied to disrupt an autosomal gene and obtain homozygous mutants in G_1_. This protocol should be suitable for the routine functional analysis of *B. mori* genes.

## Supporting Information

Figure S1 **Sequence information on pBlue-TAL TALEN scaffold and wild-type TAL effector.** (**A**) Complete nucleotide sequence of pBlue-TAL *Sal* I and *Xba* I fragment containing TALEN scaffold (NΔ152/C+63) used in this study. (**B**) Annotated amino acid sequence of the PthAp TAL effector from *Xanthomonas citri* (GenBank accession AFP97665). Amino acid numbering system used for the description of various TALEN scaffolds is based on the positions relative to the DNA binding domain (numbers preceded by a “+” mark truncations, whereas numbers preceded by a “Δ” mark deletions). RVDs are highlighted in blue. N-terminal and C-terminal amino acids used in pBlue-TAL are highlighted in yellow.
(TIF)

Figure S2 **Detection of** ***BmBLOS2*** **mutants by multiplex colony PCR.** M denotes lanes containing candidate mutant alleles detected by multiplex PCR, E – control empty vector, X – reaction failure. Circles mark mutants detected by sequence analysis. Detection by PCR failed in only six cases out of 32.
(TIF)

Figure S3 **Mutation detection in** ***Bm-re*** **gene.** (**A**) Somatic cell assay of a TALEN pair specific for *Bm-re* gene. Genomic DNA was isolated from a pooled sample of 45 microinjected embryos. The targeted DNA region was amplified by PCR and digested by the restriction enzyme *Bgl* II. The result (the right lane – “TALEN +“) revealed that a large majority of somatic cells was mutagenized. (**B**) Germline mutagenesis of *Bm-re* gene: genomic DNA was extracted from two batches of 25 eggs from nine broods (1–8 and Sib), the target region was amplified by PCR and the resulting fragments were digested with the restriction enzyme *Bgl* II. As indicated, the PCR product specific for the single brood obtained by sibling mating contained only the mutated allele, while the products from other broods contained both WT and mutated alleles.
(TIF)
