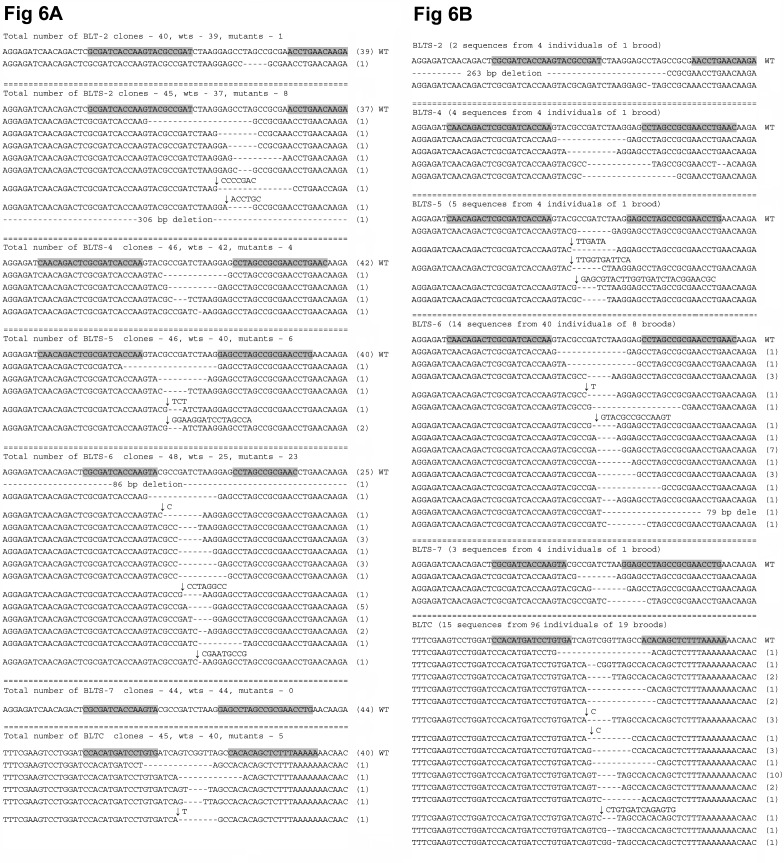# Correction: Efficient TALEN Construction for *Bombyx mori* Gene Targeting

**DOI:** 10.1371/annotation/c544ae05-f54d-488a-912a-9c4a21eb9117

**Published:** 2013-11-07

**Authors:** Yoko Takasu, Suresh Sajwan, Takaaki Daimon, Mizuko Osanai-Futahashi, Keiro Uchino, Hideki Sezutsu, Toshiki Tamura, Michal Zurovec

The arrowheads marking the phenotypes in Figure 4B are missing. Please see the correct Figure 4 here: 

**Figure:**
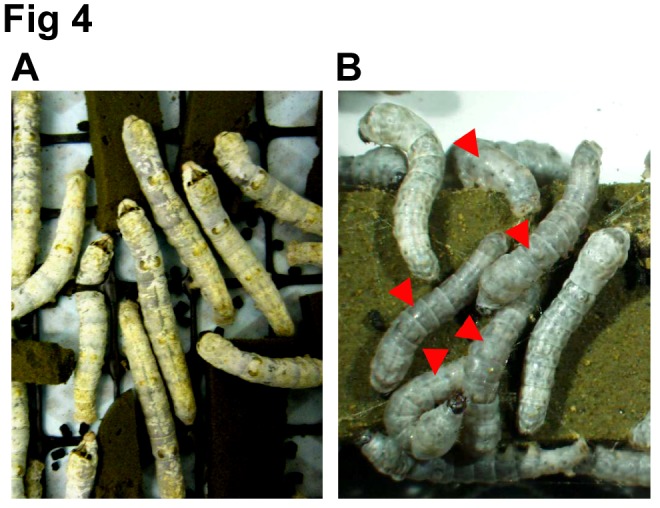


The headings differentiating Figure 6A and 6B are missing. Please see the correct Figure 6 here: 

**Figure:**