# Supplementary figures and images for: Efficient TALEN Construction for Bombyx mori Gene Targeting
Source: PLoS One. 2013 Sep 18;8(9):e73458. doi: 10.1371/journal.pone.0073458 (PMC3776831; doi:10.1371/journal.pone.0073458)

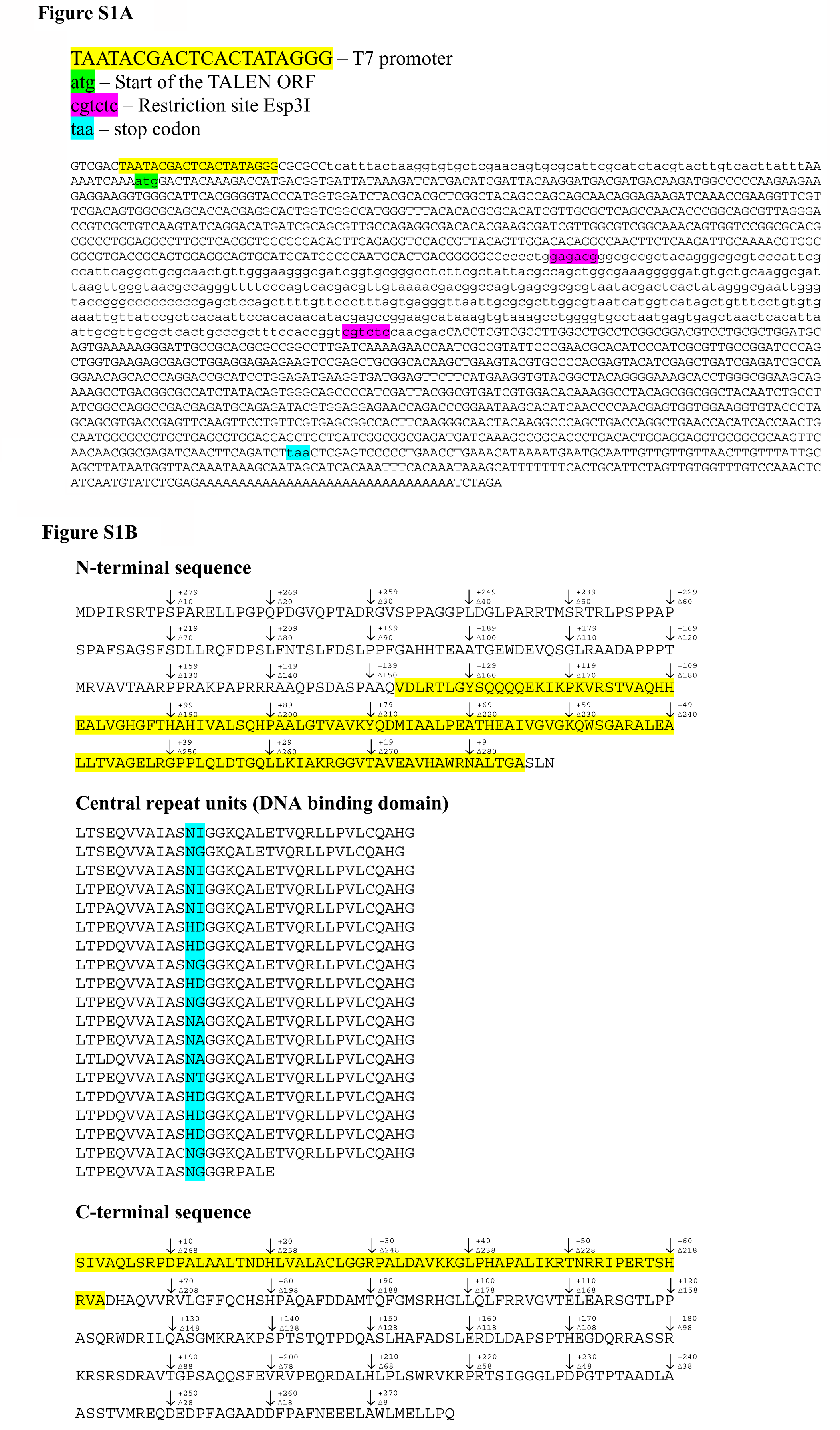

Supplement: Figure S1 — Sequence information on pBlue-TAL TALEN scaffold and wild-type TAL effector. (A) Complete nucleotide sequence of pBlue-TAL Sal I and Xba I fragment containing TALEN scaffold (NΔ152/C+63) used in this study. (B) Annotated amino acid sequence of the PthAp TAL effector from Xanthomonas citri (GenBank accession AFP97665). Amino acid numbering system used for the description of various TALEN scaffolds is based on the positions relative to the DNA binding domain (numbers preceded by a “+” mark truncations, whereas numbers preceded by a “Δ” mark deletions). RVDs are highlighted in blue. N-terminal and C-terminal amino acids used in pBlue-TAL are highlighted in yellow. (TIF) [file pone.0073458.s001.tif]

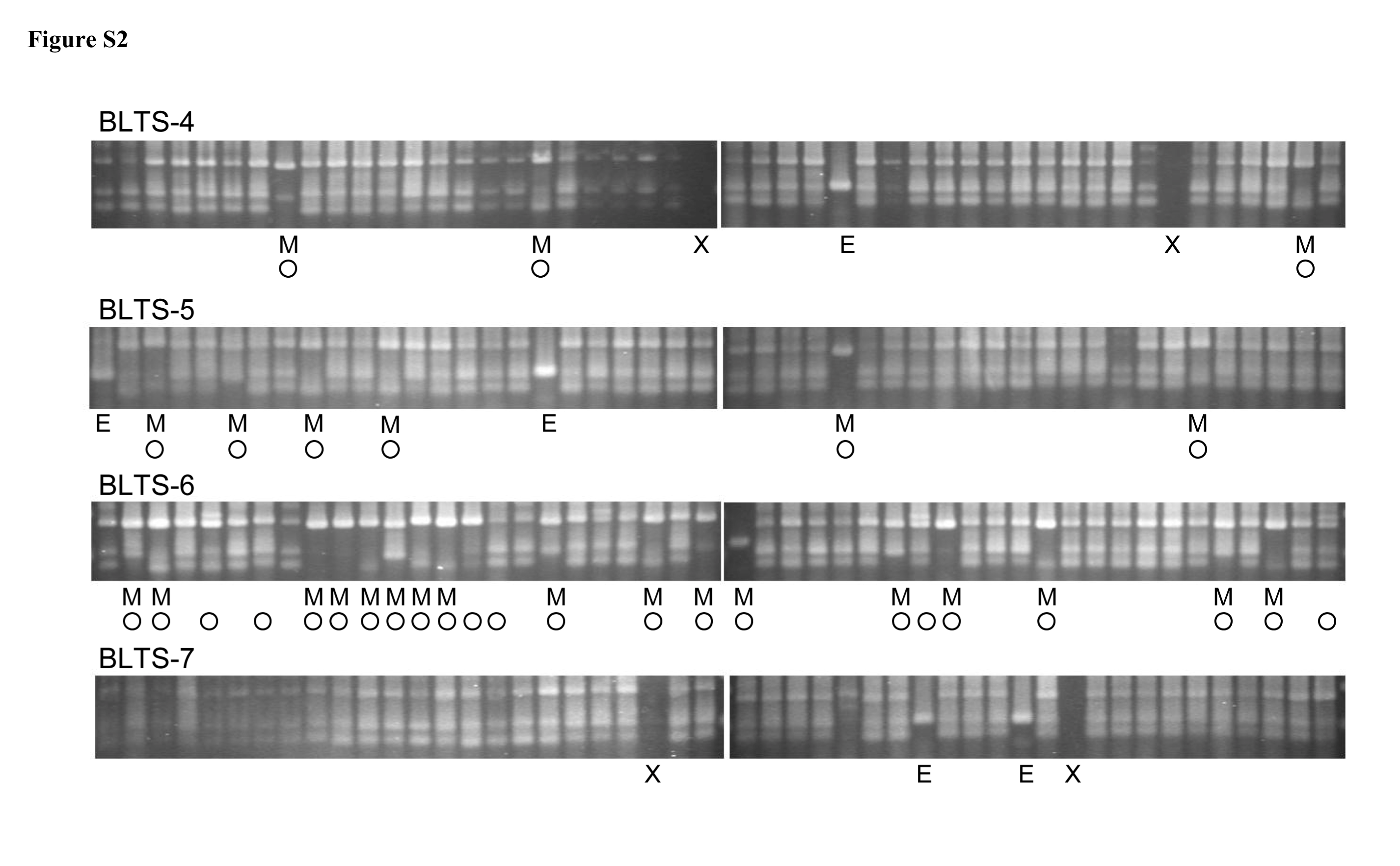

Supplement: Figure S2 — Detection of BmBLOS2 mutants by multiplex colony PCR. M denotes lanes containing candidate mutant alleles detected by multiplex PCR, E – control empty vector, X – reaction failure. Circles mark mutants detected by sequence analysis. Detection by PCR failed in only six cases out of 32. (TIF) [file pone.0073458.s002.tif]

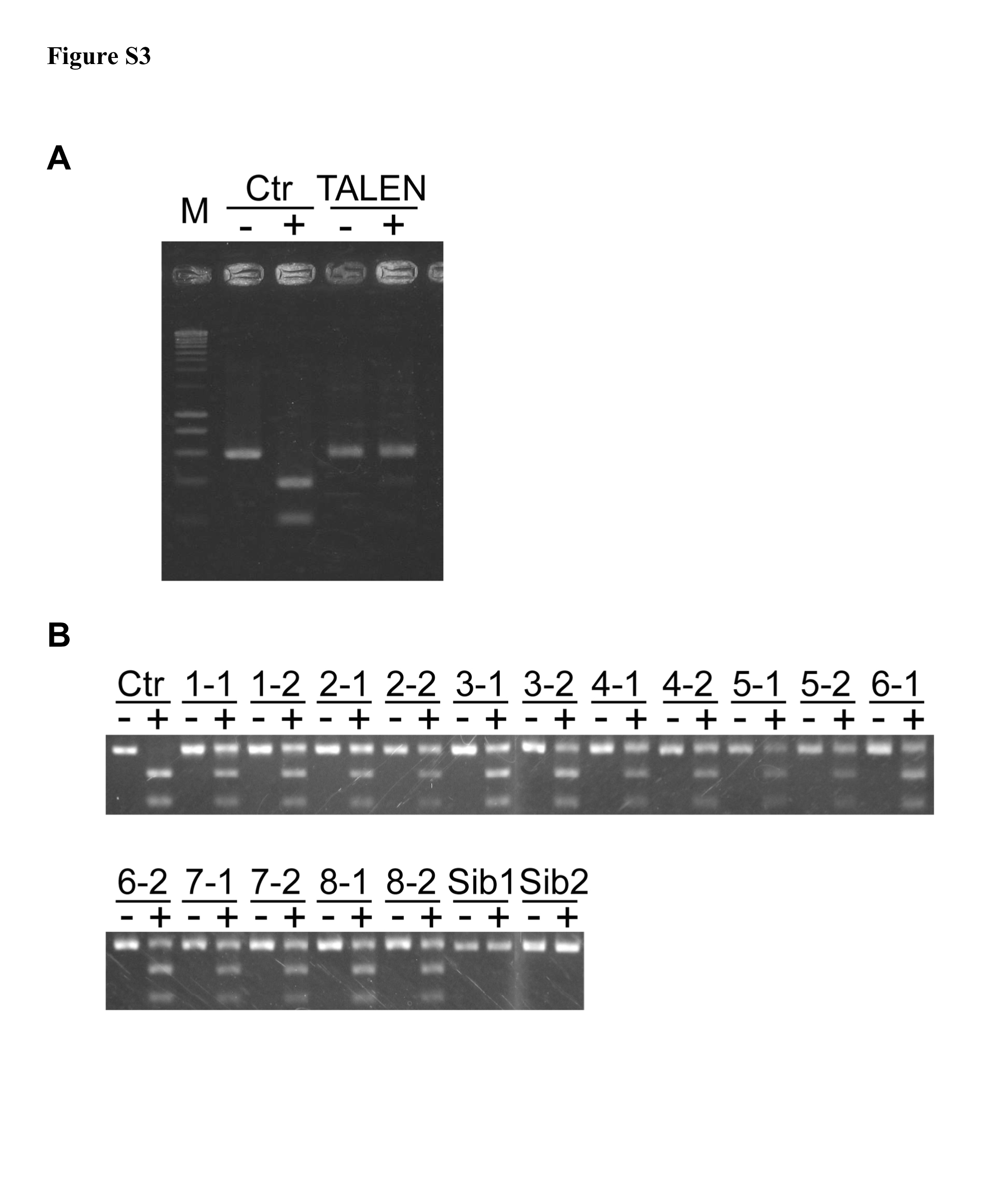

Supplement: Figure S3 — Mutation detection in Bm-re gene. (A) Somatic cell assay of a TALEN pair specific for Bm-re gene. Genomic DNA was isolated from a pooled sample of 45 microinjected embryos. The targeted DNA region was amplified by PCR and digested by the restriction enzyme Bgl II. The result (the right lane – “TALEN +“) revealed that a large majority of somatic cells was mutagenized. (B) Germline mutagenesis of Bm-re gene: genomic DNA was extracted from two batches of 25 eggs from nine broods (1–8 and Sib), the target region was amplified by PCR and the resulting fragments were digested with the restriction enzyme Bgl II. As indicated, the PCR product specific for the single brood obtained by sibling mating contained only the mutated allele, while the products from other broods contained both WT and mutated alleles. (TIF) [file pone.0073458.s003.tif]
